# Capacity for national health policy modelling, Bahrain, Kuwait, Oman, Qatar, Saudi Arabia, United Arab Emirates

**DOI:** 10.2471/BLT.25.294844

**Published:** 2026-08-20

**Authors:** Abdullah Alibrahim, Fadi El-Jardali

**Affiliations:** a Department of Industrial and Management Systems Engineering, College of Engineering & Petroleum, Kuwait University, PO Box 5969, Safat 13060, Kuwait.; b Knowledge to Policy (K2P) Center, American University of Beirut, Beirut, Lebanon.

## Abstract

Gulf Cooperation Council countries (Bahrain, Kuwait, Oman, Qatar, Saudi Arabia and United Arab Emirates) have similar governance structures, economic profiles and health system organization, creating an opportunity for collective action on health. Similarly, these countries have the same challenges including ageing populations, climate and heat risks, immigration, service delivery and disease profiles. We argue for the need to shift from opinion-driven policy discourse to computer-based, decision-focused policy analysis that is participatory, aware of inherent uncertainties surrounding decisions and context-specific. Despite areas of excellence, modelling capacity in these countries is fragmented and underresourced and models are rarely used to guide decision-making. The coronavirus disease 2019 pandemic saw very few decision-oriented models tied to local data from Gulf Cooperation Council countries. Gulf Cooperation Council models rely on outside modellers, which limits both the relevance and ownership of the findings. The coming decade will bring challenges that demand a change in how evidence is generated and applied. A structured, regionally anchored policy-modelling system can help governments test reforms, prioritize investments and engage in scenario planning before crises force reactive measures. Our paper outlines pragmatic steps towards effective health policy modelling based on four supporting pillars: (i) a regional co-produced modelling and knowledge translation strategy tied to priority decisions; (ii) embedded capacity-building across ministries, public health institutes and universities; (iii) a viable modelling stack that allows transparency, speed and reproducibility; and (iv) explicit communication and evaluation of the uncertainty of models to reduce risks within the choices made.

## Résumé

Les pays du Conseil de coopération du Golfe (Arabie saoudite, Bahreïn, Émirats arabes unis, Koweït, Oman et Qatar) présentent des structures de gouvernance, des profils économiques et une organisation des systèmes de santé similaires, ce qui offre une opportunité d’action collective en matière de santé. Par ailleurs, ces pays sont confrontés aux mêmes défis, notamment le vieillissement de la population, les risques liés au réchauffement et au changement climatique, l’immigration, la prestation de services et les profils épidémiologiques. Nous plaidons donc en faveur de la nécessité de passer d’un discours politique fondé sur des opinions à une analyse politique informatisée et axée sur la prise de décision. Cette analyse se veut participative, consciente des incertitudes inhérentes aux décisions et adaptée au contexte. Malgré certains domaines d’excellence, les capacités de modélisation dans ces pays sont fragmentées et sous-financées, et les modèles servent rarement à orienter la prise de décision. La pandémie de COVID-19 n’a donné lieu qu’à un faible nombre de modèles d’aide à la décision qui s’appuient sur des données locales provenant des pays du Conseil de coopération du Golfe. Les modèles de ces pays reposent sur des modélisateurs externes, ce qui limite à la fois la pertinence et l’appropriation des résultats. La décennie à venir apportera son lot de défis qui exigeront une évolution dans la manière de générer et d’appliquer les données factuelles. Un système structuré de modélisation des politiques, ancré au niveau régional, permettra d’aider les pouvoirs publics à tester des réformes, à hiérarchiser les investissements et à entamer une planification par scénarios avant que les crises n’imposent des mesures réactives. Cet article présente des mesures pragmatiques visant à mettre en place une modélisation efficace des politiques de santé et qui repose sur quatre piliers: (i) une stratégie régionale de modélisation et de transfert des connaissances élaborée conjointement et liée aux décisions prioritaires; (ii) un renforcement des capacités intégré dans tous les ministères, établissements de santé publique et universités; (iii) une pile de modélisation viable garantissant transparence, rapidité et reproductibilité; et (iv) une communication et une évaluation explicites de l’incertitude des modèles afin de réduire les risques liés aux choix effectués.

## Resumen

Los países miembros del Consejo de Cooperación del Golfo (Bahrein, Kuwait, Omán, Qatar, Arabia Saudí y Emiratos Árabes Unidos) tienen estructuras de gobernanza, perfiles económicos y una organización de los sistemas de salud similares, lo que crea una oportunidad para la acción colectiva en materia de salud. Además, estos países afrontan los mismos desafíos, tales como el envejecimiento demográfico, los riesgos derivados del clima y del calor, la inmigración, la prestación de servicios y los perfiles de morbilidad. Defendemos la necesidad de pasar de un discurso político basado en opiniones a un análisis de políticas informatizado y centrado en las decisiones, que sea participativo, consciente de las incertidumbres inherentes a las decisiones y específico del contexto. A pesar de contar con ámbitos de excelencia, la capacidad para la formalización de modelos en estos países está fragmentada y carece de recursos suficientes, y rara vez se usan modelos para guiar la toma de decisiones. Durante la pandemia de la enfermedad por coronavirus de 2019, hubo muy pocos modelos orientados a la toma de decisiones que estuvieran basados en datos locales de los países del Consejo de Cooperación del Golfo. Los modelos del Consejo de Cooperación del Golfo dependen de expertos externos en modelización, lo cual limita tanto la relevancia como el control sobre los resultados. La próxima década traerá consigo desafíos que exigirán un cambio en la manera en que se generan y aplican las pruebas. Un sistema estructurado de modelado de políticas con enfoque regional puede ayudar a los gobiernos a evaluar reformas, priorizar inversiones y planificar escenarios antes de que las crisis obliguen a tomar medidas reactivas. Nuestro artículo resume los pasos prácticos necesarios para lograr la formulación de modelos de políticas sanitarias eficaces basada en cuatro pilares de apoyo: (i) una estrategia de formulación de modelos coproducida a nivel regional y de traducción del conocimiento ligada a la toma de decisiones prioritarias; (ii) fortalecimiento de capacidades integrado en los ministerios, institutos de salud pública y universidades; (iii) un ecosistema de formulación de modelos viable que permita transparencia, velocidad y reproducibilidad; y (iv) comunicación explícita y evaluación de la incertidumbre de los modelos para mitigar riesgos en las decisiones tomadas.

## ملخص

توجد في دول مجلس التعاون الخليجي (البحرين، والكويت، وعُمان، وقطر، والمملكة العربية السعودية، والإمارات العربية المتحدة) هياكل حوكمة، ونظم اقتصادية، ونظم صحية متشابهة، مما يُتيح فرصةً للعمل الجماعي في مجال الصحة. وعلى نحو مماثل، تواجه هذه الدول تحدياتٍ متشابهة، تشمل تقدم السكان في السن، ومخاطر المناخ والحرارة، والهجرة، وتقديم الخدمات، وأنماط الأمراض. نحن نسلط الضوء على ضرورة التحول من خطاب السياسات القائم على الآراء إلى تحليل للسياسات يعتمد على الكمبيوتر ويركز على اتخاذ القرارات، ويتسم بالمشاركة، وعلى دراية بأوجه عدم اليقين الكامنة في القرارات، ويراعي الأوضاع المحلية. فعلى الرغم من مجالات التميز، إلا أن قدرات وضع النماذج في هذه الدول مقسمة وتفتقر إلى الموارد، ونادرًا ما تُستخدم النماذج لتوجيه عملية صنع القرار. وقد شهدت جائحة مرض كوفيد 19 عددًا قليلًا جدًا من النماذج المستندة على القرارات، والمرتبطة بالبيانات المحلية من دول مجلس التعاون الخليجي. وتعتمد نماذج مجلس التعاون الخليجي على خبراء خارجيين لوضع النماذج، مما يحد من أهمية النتائج وملكية النتائج. وسيحمل العِقد القادم تحدياتٍ تتطلب تغييرًا في كيفية ابتكار الأدلة وتطبيقها. يُمكن لنظام وضع نماذج سياسات، منظم ومُرتبط بالوضع الإقليمي، أن يُساعد الحكومات على اختبار الإصلاحات، وتحديد أولويات الاستثمارات، والمشاركة في تخطيط السيناريوهات قبل أن تؤدي الأزمات لاتخاذ تدابير لرد الفعل. توضح ورقتنا البحثية خطوات عملية نحو وضع نماذج فعّالة لسياسات الصحة، استنادًا إلى أربعة ركائز داعمة: (أ) استراتيجية إقليمية مشتركة لوضع النماذج ونقل المعرفة، مرتبطة بالقرارات ذات الأولوية؛ و(ب) بناء القدرات المُدمج في الوزارات، ومعاهد الصحة العامة، والجامعات؛ و(ج) مجموعة أدوات وضع نماذج قابلة للتطبيق، تُتيح الشفافية والسرعة وإمكانية التكرار؛ و(د) التواصل والتقييم الصريحين لعدم اليقين في النماذج، للحد من المخاطر في الاختيارات المحددة.

## 摘要

海湾合作委员会国家（巴林、科威特、阿曼、卡塔尔、沙特阿拉伯、阿拉伯联合酋长国）的治理结构、经济概况以及卫生体系架构相似，这为卫生方面的协同行动创造了机会。与此类似，这些国家也遭遇相同的难题，包括人口老龄化、气候与高温风险、移民、服务提供和疾病谱。我们主张需要从观点主导的政策话语转向基于计算机、聚焦决策的政策分析，这些分析应具备参与性，知晓决策本身具有的不确定性，同时契合各地实际情况。尽管这些国家在一些方面表现出色，但它们的建模能力分散，资源短缺，并且这些模型很少用于指导决策制定。2019 新冠肺炎疫情期间，海湾合作委员会国家几乎没有出现依托本地数据的服务决策导向模型。海湾合作委员会的模型依赖于外部建模人员，这限制了研究结论的适配性和各国对研究结论的自主掌控权。未来十年将带来各种挑战，这就要求对生成与运用证据的方式做出改变。一套结构化、依托区域的政策建模体系能够帮助各国政府在危机倒逼被动应对措施之前，测试改革方案，厘清投资优先重点，并开展情景规划。本文根据以下四大支撑支柱，概述了实现高效卫生政策建模的务实步骤：(i) 区域性协同共建的建模与知识转化策略，紧紧围绕重点决策；(ii) 在各部门、公共卫生机构及大学内部进行的嵌入式能力建设；(iii) 一套能够兼具透明性、高效率与可复现性的切实可用的建模工具栈；(iv) 明确阐释和评估模型的不确定性，以减少所做选择中的风险。

## Резюме

Страны Совета сотрудничества арабских государств Персидского залива (Бахрейн, Катар, Кувейт, Объединенные Арабские Эмираты, Оман и Саудовская Аравия) имеют схожие структуры управления, экономические профили и организацию систем здравоохранения, что создает возможности для совместных действий в сфере охраны здоровья. Эти страны также сталкиваются с одинаковыми проблемами, включая старение населения, климатические риски и угрозы, связанные с аномальной жарой, иммиграцию, предоставление услуг и профили заболеваний. Авторы считают, что необходимо переходить от дискуссий по выработке политики, основанных на субъективных мнениях, к компьютерному анализу политики, ориентированному на принятие решений, который подразумевает участие граждан, учитывает неизбежную неопределенность, связанную с решениями, и специфику конкретного контекста. Несмотря на достигнутые в некоторых областях высокие результаты, потенциал моделирования в этих странах фрагментирован, не обеспечен достаточными ресурсами, а сами модели редко используются для обоснования принимаемых решений. В период пандемии коронавируса 2019 года было создано очень мало ориентированных на принятие решений моделей, привязанных к местным данным стран Совета сотрудничества арабских государств Персидского залива. Страны Совета сотрудничества арабских государств Персидского залива полагаются на внешних специалистов по моделированию, что ограничивает как актуальность, так и чувство сопричастности к полученным результатам. Грядущее десятилетие принесет проблемы, которые потребуют изменения подходов к получению и применению доказательных данных. Структурированная, регионально адаптированная система моделирования политики может помочь правительствам тестировать реформы, расставлять приоритеты в инвестициях и заниматься сценарным планированием до того, как кризисы вынудят принимать ответные меры. В статье изложены прагматичные шаги на пути к эффективному моделированию политики здравоохранения, основанные на четырех опорных элементах: (i) региональная стратегия совместного моделирования и трансляции знаний, увязанная с приоритетными решениями; (ii) интегрированное наращивание возможностей в министерствах, институтах общественного здравоохранения и университетах; (iii) жизнеспособный стек моделирования, обеспечивающий прозрачность, скорость и воспроизводимость; (iv) четкое информирование о неопределенности моделей и ее оценка для снижения рисков в рамках принимаемых решений.

## Introduction

The six Gulf Cooperation Council states (Bahrain, Kuwait, Oman, Qatar, Saudi Arabia and United Arab Emirates) share a distinct health policy landscape. These high-income nations have among the world’s highest diabetes and obesity prevalence rates, ageing populations and climate-related health risks including heat-related mortality.^1^^–^^4^ A defining feature is their demographic structure: in four of the states, non-citizens make up most of the population, ranging from about 50% to over 85%.^5^ This structure creates distinct operational challenges as different populations face different health risks, access separate health-care pathways and move across borders with high frequency. Models assuming stable, homogeneous populations therefore misrepresent the health systems of Gulf Cooperation Council countries. Despite these complexities, policy debates too often rely on opinion, precedent or outside prescriptions rather than locally tailored analysis that accounts for this heterogeneity.^6^^–^^11^

The coronavirus disease 2019 (COVID-19) pandemic briefly put modelling on the policy agenda, but for most countries in the Gulf Cooperation Council region, modelling efforts were ad hoc, externally driven and rarely integrated into ongoing decision processes.^12^^–^^14^ Only a small number of national teams produced models fitted to local data and contexts, most of which were related to emergency response rather than long-term system planning.^12^^,^^15^ The result has been a reactive rather than proactive use of modelling: insights that arrive too late to shape policy, or that are based on assumptions that do not reflect local realities.

The next 10 years will bring challenges. Financial pressures will grow as populations age, the rate of noncommunicable diseases rises and the demand for advanced medical technologies outpaces available resources. Heatwaves, water scarcity and other climate risks are already disrupting health and livelihoods. Migration, both within and across borders, will reshape health needs and service provision.^5^ In such a context, decision-makers require tools that can synthesize different data, take account of uncertainty and make clear the trade-offs in policy choices.^16^^–^^19^

In this paper, we argue that Gulf Cooperation Council countries can no longer afford to rely only on expert opinions or fragmented, externally led modelling exercises to inform health policy. Instead, we set out pragmatic steps for developing a regional policy modelling system for health whose outputs are ready to inform specific decisions. This system would be co-produced with local stakeholders, embedded in institutions, underpinned by open and transparent technical infrastructure, and explicit about the uncertainty in its data, assumptions and projections.^16^^,^^20^^,^^21^ This shift, from opinion-driven debate to computer-based decision support, is not only feasible but essential if the Gulf Cooperation Council countries are to build resilient, equitable health systems in the face of accelerating change.

## Barriers to policy modelling

### Capacity gaps and fragmentation

The most immediate barrier is the lack of policy-oriented modelling expertise in ministries and national institutes. While many universities train scientists in mathematics, economics or public health, few offer programmes in public policy modelling and even fewer focus on the details of health policy modelling.^22^ This situation creates a mismatch: pockets of technical skills exist in isolation from policy contexts, while policy expertise is disconnected from quantitative methods. The result is a fragmented landscape where epidemiologists, economists and public health specialists work in parallel rather than through integrated modelling teams. Without structured pathways to bring these disciplines together, the region will not have the professionals who can translate complex policy questions into workable models, interpret uncertainty for decision-makers and adapt international frameworks to local contexts. This expertise gap is particularly acute in government settings, where short-term political cycles and administrative pressures leave little room for developing the long-term analytical capabilities that effective policy modelling requires.

### Data limitations

Robust models require timely, high-quality data, yet significant gaps persist in the countries’ health information infrastructure. Many Gulf Cooperation Council countries lack integrated surveillance systems, interoperable electronic health records or open-data policies that would enable comprehensive analysis. Potential political sensitivities about health data and fragmented governance structures create additional barriers to cross-sectoral and cross-border information sharing.

The COVID-19 pandemic revealed this paradox clearly. Gulf Cooperation Council countries used sophisticated digital contact tracing and surveillance systems,^23^ but the data generated from these systems was largely inaccessible to modellers due to privacy restrictions and separate institutional data repositories. Consequently, most analyses relied on publicly available aggregate statistics, which undermined model precision when decision-makers needed it most.

This data accessibility problem extends beyond emergencies. In the absence of longitudinal data sets on health expenditure, workforce dynamics and health service use, models cannot accurately capture health system responses to policy interventions. When Gulf Cooperation Council countries enact mandatory insurance or restructure health-care delivery models, the lack of integrated data systems makes it difficult to model implementation pathways, predict resource requirements or anticipate unintended consequences. Regional cooperation on data standards and coordinated investments in interoperable digital health infrastructure are hence essential for decision-ready modelling.

### Demand challenges

The main challenge is not a lack of interest of policy-makers, but a divergence between what decision-makers need and what modelling traditionally delivers. Current models often arrive after decisions have been made, present findings in technical language that obscures actionable insights, or address theoretical scenarios rather than urgent real-world policy dilemmas. When Saudi Arabia started implementing its Vision 2030 health transformation, or when the United Arab Emirates scaled up its national health insurance scheme, modelling could have revealed implementation pathways and resource requirements, yet this approach was largely absent from the public policy process.

This lack of modelling creates a vicious cycle: without exposure to decision-relevant models, policy-makers cannot communicate their modelling needs, leading to academic exercises that further reinforce scepticism about the practical value of modelling. The solution lies in demonstrating the immediate usefulness of modelling through early successes achieved by targeted analyses, designed jointly with policy-makers, that provide effective decisions addressing pressing issues within political timelines. Success breeds demand: once policy-makers experience how models can reduce the risks of major investments or reveal unintended consequences, they become advocates for expanding modelling capacity. The World Health Organization (WHO) Regional Office for the Eastern Mediterranean pioneered participatory modelling during COVID-19 by engaging policy-makers throughout the modelling process rather than delivering completed analyses to passive recipients. This approach built trust and ownership by ensuring analyses addressed country-relevant questions and fostered shared learning between researchers and decision-makers.^24^

Modelling is one tool among many, not a substitute for robust evidence infrastructure. Models are simplifications that risk false precision as outputs depend entirely on data quality and assumptions. Political actors may misuse models to justify predetermined conclusions. Alternative approaches, such as strengthened civil registration and vital statistics, rapid field epidemiology, quasi-experimental policy evaluation and descriptive analytics, are still essential complements.^16^^,^^20^ The goal is not to replace these methods but to add modelling as a tool within a broader evidence system which can be applied carefully where complexity and uncertainty warrant the investment.

## Steps to health modelling

Addressing these barriers requires a coordinated approach that builds on existing regional strengths while introducing new analytical capabilities. Rather than pursuing isolated technical solutions or capacity-building efforts, the Gulf Cooperation Council needs an integrated framework that connects institutional development, technical infrastructure, workforce training and decision-maker engagement. Fig. 1 illustrates how these four pillars operate as an integrated system, with a central coordination hub enabling both direct oversight and collaborative relationships between technical and institutional elements. This framework emphasizes that successful implementation requires simultaneous progress in all pillars rather than sequential development. The following roadmap outlines specific actions within each pillar, which are designed to deliver measurable policy impact within 3 years.

**Fig. 1:**
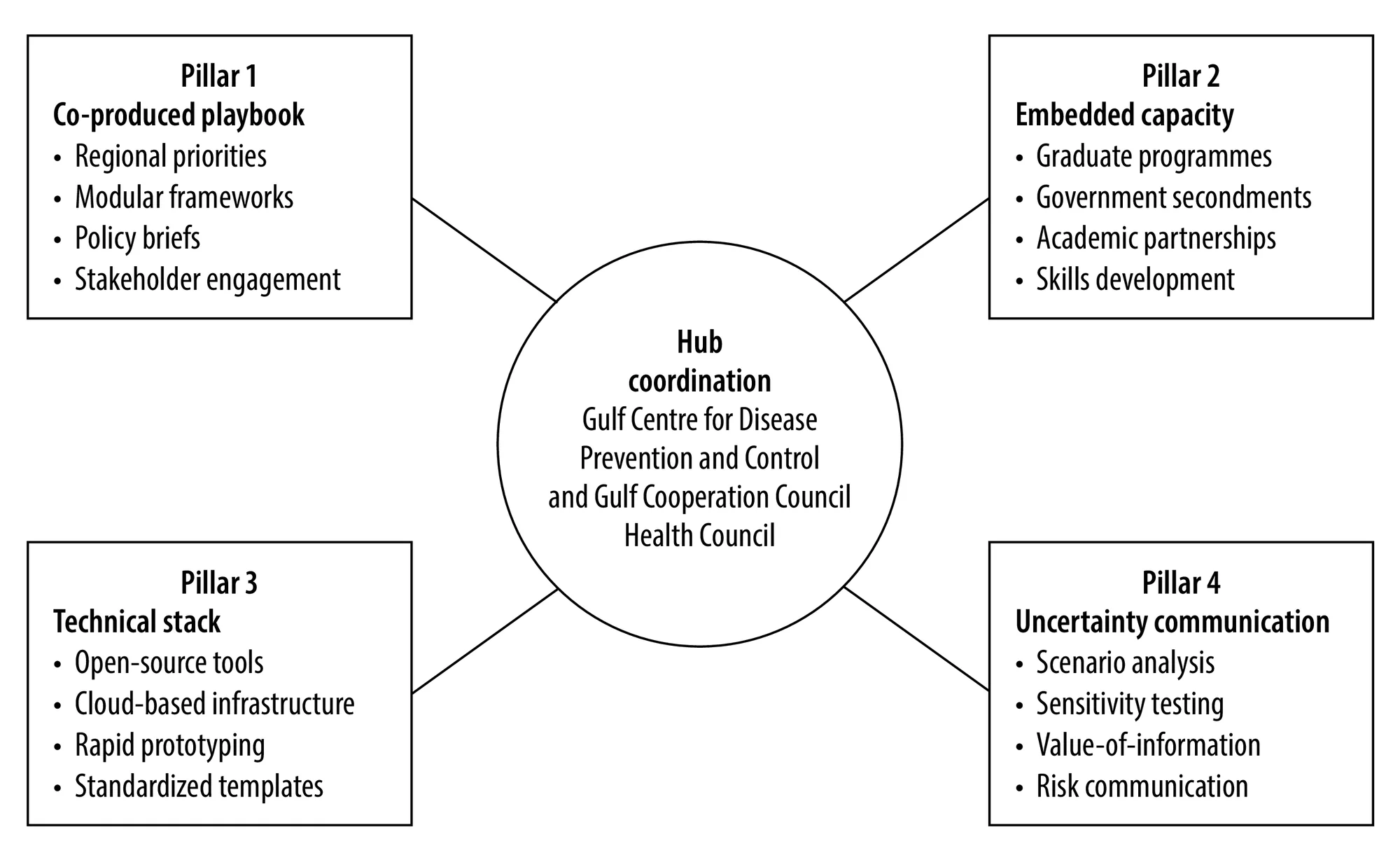
Pillars supporting health policy modelling capacity in Gulf Cooperation Council countries, Bahrain, Kuwait, Oman, Qatar, Saudi Arabia, United Arab Emirates

### Co-produced modelling strategy

The first pillar is to co-produce a modelling strategy based on regional priorities. Rather than using generic frameworks, countries should collectively identify important decision points, such as extreme-heat preparedness, health-financing reforms or mitigation of cardiometabolic disease risks, and develop modular models that can be adapted to different contexts. The process should involve ministries of health and finance, planning commissions, public health institutes and universities. The Gulf Centre for Disease Prevention and Control, established in 2021, provides an ideal institutional anchor with existing technical committees, surveillance networks and cross-border coordination mechanisms.^25^^,^^26^ Following the European Health Information Initiative model, the modelling strategy could be managed by a specialized working group within the expanding mandate of the Gulf Centre for Disease Prevention and Control.^27^

Co-production with ministries of health and finance, planning commissions, public health institutes and universities ensures that the models reflect local realities and foster ownership. Finally, translating complex modelling outputs into short policy briefs is essential. These briefs should clearly outline policy options, trade-offs and the level of certainty in the findings, enabling decision-makers to act without being overwhelmed by technical detail.

### Embedded capacity-building

Developing local modellers (the second pillar) requires sustained investment beyond short courses. Regional centres of excellence should offer graduate programmes, fellowships and reciprocal academia−government appointments. Modellers can be assigned within ministries to work on live policy questions, while mid-career public servants could take sabbaticals in academic modelling groups. These exchanges mirror the participatory approach adopted by the WHO Regional Office for the Eastern Mediterranean but extend it beyond emergencies to routine decision support.^13^

### Minimum model stack

Rather than building from scratch, the Gulf Cooperation Council should utilize proven, lightweight tools that can deliver policy insights within weeks, not months (the third pillar). The core stack, that is, the shared set of software tools, data pipelines and processes, should centre on three components: standardized data pipelines using existing health information systems; simple modelling frameworks that policy-makers can understand and examine; and rapid deployment capabilities that can run on existing government information technology systems.

Practically, this approach means adopting open-source tools such as R and Python for analysis, assisted by the proliferating large language models, and packaging them in user-friendly interfaces that ministry staff members can operate without extensive programming knowledge. The existing surveillance infrastructure of the Gulf Centre for Disease Prevention and Control provides a natural starting point, since current disease monitoring dashboards can be extended to include modelling of different policy scenarios. For example, the same systems tracking vaccination coverage could model the health and economic impacts of different immunization strategies.

For infectious disease threats, modelling must build on, not replace, surveillance, namely real-time syndromic monitoring, laboratory-based pathogen identification, genomic sequencing for variant detection and cross-border event reporting under the International Health Regulations.^28^ The regional surveillance network of the Gulf Centre for Disease Prevention and Control provides the essential infrastructure.^25^ Modelling extends these systems by projecting outbreak trajectories, estimating unreported cases and comparing intervention scenarios, but only when surveillance generates reliable inputs. The emphasis must be on speed and iteration: a working prototype deployed in 6 weeks provides more value than a comprehensive model requiring 2 years. Placing models on cloud-based platforms ensures that they remain accessible during crises, while standardized templates allow successful approaches in one country to be rapidly adapted by its neighbours. Version control and documentation standards, already familiar to the Gulf Cooperation Council’s technology sector, can make certain that models remain trustworthy and reproducible as they evolve from quick policy assessments to more sophisticated analytical tools.

### Uncertainty communication

Policy models must make uncertainty explicit (the fourth pillar). Scenarios exploring alternative assumptions, sensitivity analyses evaluating the influence of parameters and probabilistic projections all help decision-makers understand risks. Value-of-information analysis provides a formal way to quantify which uncertainties matter most and where additional data collection would produce the greatest benefit.^29^^–^^31^ Value-of-information methods treat models as decision problems, quantifying the expected benefits of eliminating or reducing uncertainty. These methods have been widely used in health-care policy-making and can guide the design of surveillance systems and studies. By integrating value-of-information and scenario analysis, policy-makers can prioritize investments in data, choose robust policies and avoid false precision.

## Opportunities for early wins

Overcoming the capacity, data and demand-side barriers described earlier requires a coordinated response that simultaneously addresses the technical, institutional and political dimensions. The Gulf Cooperation Council’s unique convergence of challenges and capabilities creates an opportunity to demonstrate the transformative potential of modelling through targeted pilot initiatives that can deliver immediate policy value.

The countries of the Gulf Cooperation Council have substantial foundations for modelling collaboration. The Hajj health card initiative successfully demonstrated cross-border health data interoperability among 250 000 pilgrims from multiple countries,^32^ while Saudi Arabia’s 50 billion United States dollars digital health investment and the United Arab Emirates’ adoption of the Internet of Things in health care provide robust technical infrastructure.^33^^,^^34^ Existing Gulf Centre for Disease Prevention and Control partnerships, field epidemiology training programmes, and improvements in the surveillance of the Gulf Centre for Disease Prevention and Control create a needed workforce and data foundations.^35^ These assets can be used immediately rather than built from scratch.

The most pressing challenges are not always the most strategic target, as the most important problems can also be the slowest to model. We therefore propose that early successes be selected on four criteria: (i) political relevance where a real decision is on the table within the next 12−18 months; (ii) data availability such that input data either exist or can be assembled in the first 6 months; (iii) cross-border applicability so results carry value across multiple member states rather than just one; and (iv) technical feasibility within a 6−12 month delivery window. Together, these criteria identify challenges that are both pressing and can be managed. Based on these criteria, noncommunicable disease prevention, and workforce and population health planning stand out as priority areas.

The diabetes and obesity epidemics in these countries necessitate models that go beyond the health ministry. Integrating data streams from food import statistics, urban planning databases, retail sales patterns and transportation usage could reveal which policies most effectively reduce the burden of noncommunicable diseases. For instance, modelling the health returns of restricting fast-food outlets near schools, implementing sugar taxes or redesigning urban environments for active transport (e.g. walking and cycling) could quantify the business case for inter-ministerial coordination.

The Gulf Cooperation Council’s non-citizen majority populations, including both expatriate professionals and labour migrants, create a need for modelling that is absent elsewhere. Predictive models must incorporate migration patterns, visa-status transitions and population heterogeneity to accurately project health-service demand.^4^ For the health workforce specifically, models balancing nationalization goals and the need for quality could show how telemedicine and task-shifting reduce dependence on expatriate specialists. For labour migrants, occupational health models addressing heat-related illness and working conditions could quantify the returns from policies for enhanced protections.^3^^,^^5^

Other areas such as health financing reform, climate-health adaptation, antimicrobial resistance surveillance and pharmaceutical supply-chain resilience satisfy the same criteria and could be included among the initial early successes or taken up soon afterwards.

These early successes share common features: they address urgent regional priorities, require cross-border coordination and promise immediate policy relevance. Success in these areas would establish modelling as indispensable infrastructure, not an optional luxury.

## Conclusion

The Gulf Cooperation Council’s current health policy landscape presents both challenges and opportunities. While Gulf Cooperation Council countries benefit from substantial financial resources and relatively cohesive governance structures, health systems face mounting pressures from demographic transitions, climate risks and a rising burden of noncommunicable diseases. These challenges demand more sophisticated analytical approaches than opinion-based policy discourse can provide.

The steps outlined in our paper build on existing strengths, such as the Gulf Centre for Disease Prevention and Control’s coordination mechanisms, substantial digital health investments and demonstrated capacity for cross-border collaboration, rather than requiring new institutional frameworks. The four pillars work together: (i) co-production ensures models reflect local realities; (ii) embedded capacity-building sustains the skills to maintain these models; (iii) a shared minimum model stack keeps analyses fast, transparent and reproducible; and (iv) explicit communication of uncertainty, through short policy briefs outlining options, trade-offs and the level of certainty in the findings, enables decision-makers to act without being technically overwhelmed.

Implementation will need to proceed incrementally, in step with institutional readiness and political commitment rather than on a rigid timetable. A possible first phase could focus on one or two of the priority areas identified above, with at least one area having a cross-border form to demonstrate the value of coordination. In the following year, a second phase would aim to embed modelling capacity through fellowships, secondments and reciprocal academia−government appointments. During this phase, countries would also advance the data arrangements that the early successes require and train initial cohorts of government-embedded modellers. Over a 3-year horizon, the realistic goal is for modelling to begin informing policy decisions, such as sugar-tax calibration, fast-food zoning near schools or task-shifting protocols that reduce dependence on expatriate specialists, and to provide evidence for sustained investment. The exact pace and scope will depend on funding, member state interest and the willingness of existing institutions to host the work. Early successes are not stand-alone deliverables but the first step towards an embedded modelling ecosystem.

The economic case for investment in modelling is compelling: improved policy targeting through evidence-informed analysis can generate cost savings that exceed infrastructure investments within the first implementation cycle. More importantly, the analytical capabilities developed for health policy can be extended to other sectors facing similar uncertainty and complexity.

The Gulf Cooperation Council possesses the resources, governance capacity and innovation culture to lead this transformation. Success in building regional modelling capacity will depend on sustained political commitment, adequate funding mechanisms and willingness to adjust the approach based on early experience. The Gulf Cooperation Council’s track record in large-scale coordination and innovation suggests these conditions can be met, making this transition from opinion-driven to evidence-informed health policy both achievable and essential.
